# THE SENIOR COMMUNITY SERVICE EMPLOYMENT PROGRAM: NEW DATA ON OLDER ASIAN WORKERS

**DOI:** 10.1093/geroni/igac059.3077

**Published:** 2022-12-20

**Authors:** Patrick Ho, Lam Lai, Cal Halvorsen

**Affiliations:** Boston College, Boston, Massachusetts, United States; Boston College, Boston, Massachusetts, United States; Boston College, Allston, Massachusetts, United States

## Abstract

The Senior Community Service Employment Program (SCSEP) is the only federal employment program for people aged 55 and older. While 6% of the approximately 60,000 participants are Asian and federal funds are set aside to serve Asian workers, little is known about their personal characteristics and experiences in SCSEP. Using novel data from an online and paper-based Massachusetts survey of SCSEP participants from April to August 2022 offered in multiple languages, this study aimed to describe Asian participants’ characteristics and experiences in SCSEP. Respondents (Nf39) ranged in age from 58 to 73. Almost all spoke a language other than English at home, and all respondents were born outside of the U.S. Nearly half reported a high school degree or less, and none reported “excellent” health. One-third reported feeling lonely occasionally or in specific situations. While two-thirds have made recent tradeoffs in paying for important goods and services (e.g., food and health care), most reported that their personal finances, social engagement, family life, and self-confidence have improved due to SCSEP. Further, 9 in 10 agreed that their supervisors understood the goals of SCSEP and were supportive. A quarter said separately that it was very likely they would search for a paid job or volunteer role after exiting SCSEP. These results contribute to the small amount of literature on older Asian American workers while informing the work of organizations that serve older Asian workers who have experienced multiple barriers to employment relating to nativity status, language abilities, and anti-Asian discrimination.

